# Acute respiratory distress syndrome - the Berlin definition: impact on an ICU of a university hospital

**DOI:** 10.1186/cc12032

**Published:** 2013-03-19

**Authors:** A Agrifoglio, J Lopez, J Figueira, M Hernández, L Fernández, M Irazabal, S Yus, M Jiménez

**Affiliations:** 1 Hospital Universitario La Paz, Madrid, Spain

## Introduction

The current definition of ARDS was established in 1994 by the AECC. In 2011 emerged the Berlin definition. The aim of this study was to find a new approach from the impact of ARDS in an ICU and to analyze APACHE II score, days of mechanical ventilation (MV), duration of ARDS and mortality.

## Methods

We analyzed the database of our ICU collected prospectively from October 2008 to January 2011 and regarded all patients undergoing MV for 48 hours or more and who met the AECC criteria. All patients were ventilated according to the ARDSNet protocol.

## Results

During this period 1,900 patients were admitted, 607 were under MV for at least 48 hours and 104 had ARDS criteria. Moderate ARDS 60.6%: 60% male, median age 53 years, APACHE II mean 22.49 (95% CI = 20.71 to 24.27), mean duration MV 23.84 (95% CI = 18.08 to 29.60), ARDS duration 12.87 (95% CI = 9.83 to 15.90), average stay 29.22, mortality 52.4%. Severe ARDS 39.4%: 63% male, median age 45 years, APACHE II mean score 23.19 (95% CI = 20.62 to 25.75), mean duration MV 18.52 (95% CI = 13.01 to 24.03), ARDS duration 12.15 (95% CI = 8.72 to 15.58), average stay 22.65, mortality 48.8% with no statistically significant difference with moderate ARDS (*P *= 0.72). Leading cause of death in both groups: multiorgan dysfunction (60%). See Tables 1 and 2.

**Table 1:** Subgroup analysis: moderate ARDS

Variable	Survivors	Nonsurvivors	*P *value
APACHE II	20.06 ± 6.96	24.83 ± 6.17	0.006
MV (days)	25.63 ± 29.13	22.21 ± 15.43	0.557
ARDS (days)	10.29 ± 9.07	15.20 ± 12.92	0.107
ICU stay	34.33 ± 33.26	24.57 ± 16.91	0.142

**Table 2:** Subgroup analysis: severe ARDS

Variable	Survivors	Nonsurvivors	*P *value
APACHE II	20.82 ± 8.89	25.31 ± 5.63	0.761
MV (days)	26.95 ± 18.03	10.10 ± 11.64	0.001
ARDS (days)	15.8 ± 11.60	8.5 ± 8.6	0.031
ICU stay	33.52 ± 18.25	11.25 ± 11.80	0.001

## Conclusion

In this new approach of our ARDS patients we found no statistically significant differences in relation to the variables of interest to analyze in the two groups. Patients with severe ARDS who survive ICU admission have a longer duration of ARDS, which means more days on MV and therefore increased ICU stay.
